# Outcomes of the Pregnancies with Chronic Myeloid Leukemia in the Tyrosine Kinase Inhibitor Era and Literature Review

**DOI:** 10.3390/hematolrep14010008

**Published:** 2022-03-20

**Authors:** Dan Ran Castillo, Daniel Park, Akhil Mehta, Simmer Kaur, Anthony Nguyen, Mojtaba Akhtari

**Affiliations:** 1 Department of Hematology/Oncology, Loma Linda University Health, Loma Linda, CA 92354, USA; SimmerKaur@llu.edu (S.K.); makhtari@llu.edu (M.A.); 2 Internal Medicine, UCSF Fresno, Fresno, CA 93701, USA; daniel.park2@tu.edu (D.P.); alnguyen2@gmail.com (A.N.); 3 Internal Medicine, Loma Linda University Health, Loma Linda, CA 92354, USA; amehta@llu.edu; 4 Moores Cancer Center, University of California, San Diego, CA 92037, USA

**Keywords:** outcome, pregnancy, chronic myeloid leukemia, literature review

## Abstract

Chronic myeloid leukemia (CML) is a clonal myeloproliferative neoplasm (MPN) that accounts for 10% of pregnancy-associated leukemias. The Philadelphia chromosome balanced translocation, t (9:22) (q34; q11.2), is the classic mutation seen in CML. The BCR-ABL oncoprotein encoded by this mutation is a constitutively active tyrosine kinase. Tyrosine kinase inhibitor (TKI) therapy is considered a first-line treatment for CML. However, the literature has revealed risks of teratogenicity with TKI therapy during pregnancy. Understanding the risks and benefits of TKI therapy and alternative therapies such as interferon-alpha (IFN-α) will help clinicians and pregnant patients develop a personalized CML treatment plan. This manuscript presents a case series detailing the management of five pregnancies in two pregnant patients with CML and a literature review of CML management in pregnancy.

## 1. Introduction

**Figure 1:**
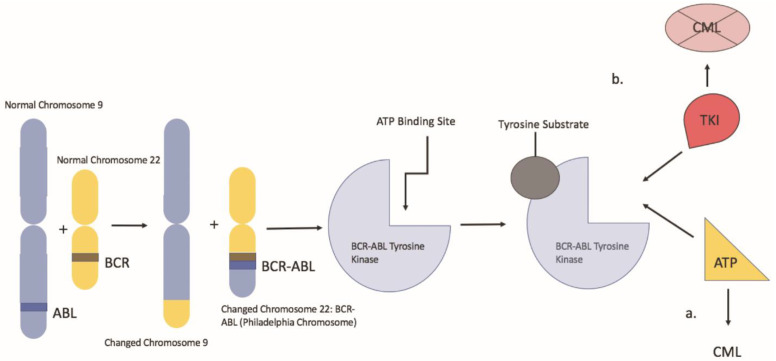
Represents a depiction of the Philadelphia chromosome balanced translocation and its mechanism of action. **a**: the BCR-ABL oncoprotein with a molecule of adenosine triphosphate (ATP) in the kinase pocket. The substrate is activated by the phosphorylation of tyrosine residues and can activate other downstream pathways. **b**: when TKI occupies the kinase pocket, the action of BCR-ABL is inhibited, preventing phosphorylation of its substrate.

Chronic myeloid leukemia is a clonal myeloproliferative neoplasm characterized by neoplastic proliferation of myeloid cells, particularly granulocytes. The hallmark of CML is the Philadelphia chromosome, which results from a reciprocal translocation between the long arms of chromosome 9 and 22, t (9:22) (q34; q11.2) (Figure 1). In this balanced translocation, the ABL1 gene of chromosome 9 (q34) is juxtaposed to the BCR gene (q11) of chromosome 22 [1]. The BCR/ABL1 fusion oncogene encodes the BCR-ABL oncoprotein, a constitutively active tyrosine kinase, which drives the proliferation of hematopoietic cells. That said, imatinib changed the landscape of CML treatment as it was the first drug designed to target the BCR-ABL tyrosine kinase protein. Imatinib works by binding the ATP binding site of the BCR-ABL oncoprotein, locking it into a closed conformation, thereby preventing the transfer of phosphate to tyrosine residues [2]. That said, despite its effectiveness, a review of the literature has revealed the risk of potential teratogenic side effects associated with imatinib use during pregnancy. This presents a significant issue for CML management given about 7.5–12% of CML patients are less than 29 years of age at the time of diagnosis in western countries and the median age of diagnosis in emerging countries is between 30–40 years; thus, in the childbearing age [3]. In these cases, alternative therapies such as pegylated IFN-α can be considered. In addition, leukapheresis is an alternative in the setting of a high white blood cell count. Understanding the risks and benefits of TKI therapy vs. alternative therapy in pregnant patients with CML will aid clinicians and pregnant patients in developing an appropriate individualized CML treatment plan. 

## 2. Case Presentations

Case 1: A 25-year-old female G1P0 without significant medical history had leukocytosis on prenatal lab review at 12 weeks. Her complete blood count (CBC) was significant for WBC 75.8 × 10^9^/L, Hemoglobin (Hgb) 11.3 g/L, and platelets (PLT) 1026 × 10^9^/L. Shortly after that, during her 14th week of pregnancy, the patient was admitted for nausea and vomiting. Her CBC on admission was significant for leukocytosis WBC 90 × 10^9^/L, Hgb 10.9 g/L, and thrombocytosis PLT 1069 × 10^9^/L. The peripheral smear showed marked leukocytosis with absolute neutrophilia and left-shifted myeloid maturation. A 100-cell differential count showed: 1% promyelocytes, 12% metamyelocytes/myelocytes, 70% segmented neutrophils, 5% lymphocytes, 2% monocytes, 0% eosinophils, and 3% basophils. Bone marrow aspirate and core marrow biopsy showed no increase in blasts (<2%) or evidence of marrow fibrosis (MF 0 on a scale of 0 to 3). Chromosome analysis revealed 46, XX, t (9,22) (q34; q11.2) (19)/46, XX (1). Subsequent fluorescence in situ hybridization (FISH) was abnormal for BCR/ABL1 rearrangement. The quantitative level of BCR/ABL1 e13/e14-a2 transcripts was >55% of the International Scale (IS), using an RT-qPCR (IS) assay with sensitivity of ≥4.5 log below the standardized baseline [4,5], and there was no JAK2/V617F mutation, consistent with CML in the chronic phase. A fetal echocardiogram completed in the second trimester demonstrated a dichorionic diamniotic intrauterine pregnancy consistent with dates, normal amniotic fluid, and no specific fetal anomalies identified. The oncology team discussed treatment options, associated risks, and benefits with the patient and her husband. Ultimately, the patient and her husband elected for pegylated IFN-α (Pegasys), requiring less frequent administration and better tolerability. With that in mind, during her 18th week of pregnancy, the patient was started on a weekly Pegasys 90 mcg injection which was gradually increased to 180 mcg. She was encouraged to continue baby aspirin, present for weekly labs, and return to her hematologist every two weeks. Two months after the patient started treatment, her leukocytosis, thrombocytosis, and neutrophilia had improved significantly (supplemental Figure S1). The patient was tolerating weekly Pegasys injections 180 mcg well and remained asymptomatic. Her course was complicated by an insurance lapse, during which time the patient’s treatment was held for four weeks, resulting in a temporary increase in WBC count, but after her Pegasys was resumed, the patient’s labs improved (Supplementary Figure S1). The patient also developed mild hypothyroidism secondary to Pegasys utilization. Her hemoglobin in the second and third trimesters remained stable at approximately 10.5–11 g/L. Ultimately, the patient had healthy twins, naturally delivered at 35 weeks and 5 days, without abnormalities noted at two-month follow-up. After delivery, the patient was started on dasatinib and she achieved a complete hematological response within six weeks.

Case 2: A 30-year-old female with no prior medical history was diagnosed with Philadelphia chromosome-positive CML in a chronic phase at 22 years of age. The patient was briefly started on hydroxyurea and then switched to the first-generation TKI (Imatinib) at a dose of 400 mg daily and achieved molecular response (MR3). Three years after her CML diagnosis, the patient experienced molecular relapse, with the percentage of BCR-ABL/ABL transcripts rising to 37.9%. She underwent a bone marrow aspiration and biopsy, which revealed a normocellular marrow (50–60 cellularity) with granulocytic left-shift and hypolobated megakaryocytes with less than 5% blasts. Cytogenetic analysis showed an abnormal female karyotype with 46, XX, t (9,22) (q34; q11.2) (13)/46, XX (7) The patient was started on a second-generation TKI (dasatinib) and subsequently achieved major molecular remission (MMR, BCR-ABL/ABL transcripts ≤ 0.1%). Two years later the patient had a planned pregnancy and dasatinib was discontinued several months prior to conception. The status of her hematological/molecular status is unclear at this point due to lack of medical follow-up. Hydroxyurea was initiated during her pregnancy to manage her leukocytosis. The patient delivered a healthy infant at 34 weeks via a scheduled cesarean section. After the delivery, she was restarted on dasatinib and achieved MMR.

Two years later, while on dasatinib therapy and in MMR, the patient became unintentionally pregnant. Ultrasonography confirmed a dichorionic diamniotic intrauterine pregnancy with an estimated 12-week gestation. Upon confirmation of pregnancy, dasatinib was discontinued and she was transitioned to IFN-α injections. Like her prior pregnancy, non-compliance with medical follow-up was encountered; therefore, surveillance ultrasound is missing. At week 12 of pregnancy, her BCR-ABL/ABL ratio was 2.6% and increased to 96.5% by week 27, with a concomitant rise in her WBC count to 31 × 10^9^/L. As a result, her IFN-α dose was increased from 3 million units to 5 million units three times a week. Unfortunately, the patient had notable side effects from the medication, including malaise, rhinitis, and nausea. At 34 weeks and 5 days, the patient had a scheduled primary low transverse cesarean section delivering a baby boy weighing 2.1 kg and a baby girl weighing 2.4 kg with Apgar scores of 9 and 9 at 1 and 5 min, respectively. The girl was born premature but otherwise healthy without any abnormalities noted at two-month follow-up. However, the male infant had a significant posterior maligned ventricular septal defect, a bicuspid aortic valve with mild hypoplasia of the ascending and transverse aorta, and a moderate size patent foramen ovale. He subsequently underwent pulmonary artery banding and patent ductus arteriosus ligation.

## 3. Discussion

CML accounts for up to 10% of pregnancy-associated leukemias, with an annual incidence of 1 per 100,000 pregnancies, with thrombosis being the most common cause of maternal morbidity. [6] That said, given more than 50% of pregnancies in leukemia patients are unplanned, management of CML during pregnancy should be individualized.

To date, there are no established guidelines for CML management in pregnancy, although some expert recommendations have been published and recently updated [7]. There are several scenarios to consider concerning CML and pregnancy such as CML discovered during pregnancy, pregnancy discovered during TKI treatment, and planned pregnancy after remission [8]. Close observation might be an option for select patients with minimal CML clinical manifestations [9]. However, whether a patient can wait or not depends on the disease stage, initial blood counts, and expected time to delivery. The first case highlighted two essential considerations, namely the potential for thrombosis due to significant thrombocytosis and leukostasis due to leukocytosis above 100 × 10^9^/L. Moreover, the second case showcased congenital defects that could occur during the critical period of organogenesis in a pregnant female taking dasatinib during the first trimester [10].

Several case series and reports have shown pregnant women who remain on TKI [11] treatment achieve molecular and hematological remissions. We summarize the recent studies on the outcome of pregnancies associated with TKI in Table 1. Pye et al. reported a study consisting of 180 pregnancies in which 70% of cases were exposed to a first-generation TKI (imatinib) during their first trimester. In total, 26% stayed on therapy during their pregnancies. Of these, congenital abnormalities were identified in 7% of cases, 10% ended up having a spontaneous abortion, and 20% were terminated by elective abortion [12,13]. Abruzzese et al. reported a group of women with a total of 265 pregnancies who were exposed to imatinib during organogenesis (>5 wks gestation). A total of 17% and 9% of cases ended up in spontaneous abortion and elective abortion, respectively. Congenital abnormalities were found in 6% of cases. Cortes et al. summarized pregnancy-related outcomes in 78 dasatinib-treated women. Pregnancies were terminated in 57% of cases; 23% and 17% of cases had elective abortions and spontaneous abortions, respectively. Nine percent of babies were found to have congenital defects and 19% of mothers delivered live normal births. Intrauterine growth restriction (IUGR) and premature deliveries were observed as maternal issues [10]. Nilotinib, another second-generation TKI, has been found to have limited and conflicting evidence regarding its use in pregnancy based on case reports. Even after nilotinib was discontinued in the first trimester once pregnancy was confirmed, an ultrasound identified a significant omphalocele in the third month. [14]. As above in our case series, case 1 demonstrates a positive outcome in pregnancy with appropriate planning and mutual decision making between physician and patient. Our CML patient was managed effectively with pegylated IFN-α, and after pregnancy achieved complete hematological response with TKI. Conversely, case 2 corroborates the risks of TKI use during pregnancy, consistent with the evidence noted in the studies. Our case 2 patient became unintentionally pregnant while on dasatinib, and even with cessation of TKI use, one of her twin children developed congenital abnormalities (posterior maligned ventricular septal defect, bicuspid aortic valve, and moderate size patent foramen ovale).

That said, the teratogenicity of imatinib is reported to be due to off-target, most likely inhibition of the mast/stem cell growth factor receptor (c-kit), platelet-derived growth factor (PDGFR) during organogenesis [15]. The second-generation TKIs also affect multiple receptor tyrosine kinases, including the proto-oncogene c-Src and the ephrin receptor kinases [16]. The literature suggests that children born to men taking imatinib at conception did not increase the risk for congenital malformations [17]. Although imatinib has been used safely in the second and third trimesters, insufficient experience does not allow routine use [18,19]. Especially for female patients, the occurrence of hydrops fetalis with dasatinib use was increased in the second trimester [20], implying dasatinib should be stopped immediately once pregnancy is identified due to high teratogenic potential in both early and late gestation. For unplanned pregnancy following CML diagnosis, fetal ultrasonography should be performed immediately. The risks and benefits of ceasing or transitioning CML-driven treatment while continuing pregnancy should be fully addressed. Some institutions suggest considering cases individually during late pregnancy since some TKIs such as imatinib and nilotinib are felt not to cross the placenta and could be considered after the 16th week [13]. Dasatinib should not be utilized at any pregnancy stage due to the increased placental transfer percentages and preclinical/clinical teratogenicity. Additionally, some researchers from the literature state that bosutinib has the highest theoretically calculated passive placental transfer rate [21]. Based on currently available studies, it is advised to avoid bosutinib during pregnancy.

It remains a pitfall for established CML patients to elect to proceed with pregnancy while on TKI treatment. For patients who had treatment with TKI, Table 2 summarizes recent studies using alternative approaches. Balsat et al. presented a series of 12 successful pregnancies in 11 CML BCR-ABL positive CML patients, whose leukemia was all managed with IFN alpha. All children had normal growth and development [22]. Chelysheva et al. evaluated a database that reported 33 patients with CML and pregnancy in the chronic phase [23]. Of these patients, 15% were treated with IFN-α and 3% had hydroxyurea. However, 39% of cases still received TKI, 42% of patients elected for no therapy, and no birth defects were observed. The outcomes for all 28 pregnancies from a study by Alizadeh et al. are summarized in Table 2. There were three early spontaneous abortions reported. All women received IFN-a during these pregnancies, and two were exposed to imatinib early in pregnancy. Twenty-three out of twenty-five babies were healthy. Congenital defects were found in two infants, although one had an insignificant ventricular septal defect, and one, who had been exposed to imatinib early in pregnancy, had a diaphragmatic hernia [24].

Outcomes for CML patients have significantly improved after the first TKI, imatinib, was approved in 2001. Recent clinical trials have addressed the possibility of discontinuing TKI treatment (treatment-free remission, TFR) [25,26]. The evidence for a prolonged TFR after TKI discontinuation originates from multiple prospective studies, which show the feasibility of a sustained molecular response after first-line TKI discontinuation in approximately 40% of patients [27,28]. There are rising numbers of publications about whether the women eligible for a trial of TFR can also safely discontinue their TKI before conceiving. Management after that depends on the maintenance or loss of major molecular response (MMR) [29]. Women who lose MMR and are not yet pregnant should restart the treatment, perhaps with a more potent TKI, and could try to discontinue again after sustained MMR has been reestablished and maintained appropriately. Conception while on active TKI therapy is in general discouraged due to the risk of fetal abnormalities, as reported in our case series. TKI therapy should be stopped before natural conception, and the patient should remain off treatment during pregnancy if possible. A more challenging condition is the woman desiring pregnancy without a sustained MMR. Other solutions include substituting TKI with IFN-a or referring to alternative methods for conception, including in vitro fertilization (IVF) centers [30].

Before the BCR-ABL TKIs, IFN-α was the treatment of choice for patients who were not candidates for transplantation [29] since IFN-α does not cross the placental barrier with its high molecular weight (19 kDa). Treatment with IFN-α adequately controls the leukemic cell mass in most newly diagnosed patients with CML by (1) selective toxicity against the leukemic clone, (2) enhancement of ‘immune’ regulation, and (3) modulation of bone marrow microenvironmental regulation of hematopoiesis. [31] IFN-α has produced complete hematologic response rates ranging from 50–80% and cytogenetic response rates ranging from 20 to 60%, with 27% achieved a complete cytogenetic response. Among the 30–50% of patients with low-risk disease, significant cytogenetic response rates have ranged from 40–50%, and median survival times from 80 to 105 months. [32].

During pregnancy, IFN-α remains the best therapeutic option [22,33,34]. It is safe for pregnant women as it is undetectable in fetal blood. However, its use is limited by the slow time to response. With that in mind, a polyethylene glycol molecule was attached to IFN-α to increase its half-life and lower immunogenicity. The resulting pegylated IFN-α (PegIFNα) is available in two commercial forms, PegIFNα-2a (Pegasys) and PegIFNα-2b (PegIntron). These two variants can be administered less frequently than unpegylated IFN-α. Pegasys is rated as a pregnancy Category C therapeutic since there are no well-controlled large-scale studies in pregnant women. However, prior publications have shown efficacy and safety in pregnant women diagnosed with myeloproliferative disease [35]. Based on our experience, it is safe to administer and efficient during pregnancies.

As there is a heterogeneous response in the CML response to IFN- α, an evolving understanding of genetics provides perspective into the variability of response. A study by Lindgren et al. has shown an association between a genetic polymorphism near the IL28B (IFNL3) gene and the response to IFN-α [36]. In particular, the presence of the genotype, *rs12979860,* was found to have a significant association with patients achieving complete response in MPN. Kriel et al. studied 174 patients with CML and treated with IFN-α [37]. Of these patients, 79 achieved less than 35% Ph positive metaphases (responders) and 95 failed to show any cytogenetic response (non-responders). Seventeen single nucleotide polymorphisms (SNPs) were compared at *IFNAR1, IFNAR2, JAK1, TYK2, STAT1, STAT3*, and *STAT5a/b*. A significant difference in minor allele frequency was seen with only one SNP (*rs6503691*). Real-time quantitative polymerase chain reaction (Rt-PCR) revealed a significant correlation between *rs6503691* and levels of *STAT3* mRNA. This was notable as BCR-ABL is known to activate STAT3, and SOCS3, a known target of STAT3, confers IFN-α resistance. The authors concluded that their results indicated that polymorphic variations in *STAT3* expression may be a determinant in the response of IFN- α to CML. With increased research into SNPs and their role in CML therapy, there will be a greater understanding of individualizing CML management.

Leukapheresis is helpful during pregnancy but only in the case of hyperleukocytosis. Hydroxyurea is usually not a safe option and is generally not indicated because of the potential teratogenic effects observed across many animal species. As a result, it is only considered appropriate after the second trimester [38], and it is typically reserved for pulse dosing to control high WBC counts [39]. In addition, the literature has suggested that selected TKIs, specifically imatinib and nilotinib, may be considered after organogenesis.

The latest CML treatment progress has significantly improved patients’ overall survival and provides most patients with a durable MMR and quality of life [40]. However, the therapeutics of CML during pregnancy is still institution/experience-based, requiring close coordination with obstetric and neonatology colleagues to satisfy maternal and fetal needs. Many publications, including the recommendations abovementioned, suggest stopping TKI early during pregnancy (3–5 weeks) to reduce the risks of relapse within the first trimester and, if possible, the pregnancy should be drug-free.

The first case highlighted two essential considerations, namely the potential for thrombosis due to significant thrombocytosis, and leukostasis due to leukocytosis above 100 × 10^9^/L. Moreover, the second case showcased congenital defects that could occur during the critical period of organogenesis in a pregnant female taking dasatinib during the first trimester.

As above in the case series, case 1 demonstrates a positive outcome in pregnancy with appropriate planning and mutual decision making between physician and patient. Our CML patient was managed effectively with pegylated IFN, and after pregnancy, complete hematological response was achieved with TKI. Conversely, case 2 corroborates the risks of TKI use during pregnancy, consistent with the evidence noted in the studies. Our case 2 patient became unintentionally pregnant while on dasatinib, and even with cessation of TKI use, one of her twin children developed congenital abnormalities (posterior maligned ventricular septal defect, bicuspid aortic valve, and moderate size patent foramen ovale).

In summary, our case series and literature review show that pregnant women with CML can be managed safely without TKI. However, this approach might not fit all patients. Full awareness of CML-directed therapy and potential risks during pregnancies will help clinicians better support patients and their families. A multidisciplinary team is warranted for the best benefit of the patients and their offspring. Although current management of CML during pregnancy is primarily empirical, patients need to be informed extensively about their risks. A treatment decision should include maternal disease status, fetal safety, and choices.

## Figures and Tables

**Table 1:** Pregnancy outcomes in patients associated with TKI therapy.

Study	n	Treatment during Pregnancy	Elective Abortion	Spontaneous Abortion	Fetal Abnormalities	Normal Live Birth	Unknown Outcome
Pye et al.	180	1st trimester Imatinib use: 70% 1st-3rd trimester Imatinib use 26%	19%	10%	7%	35%	31%
Abruzzese et al.	265	Exposed to Imatinib during organogenesis (>5-week gestation)	17%	9%	6%	48%	21%
Cortes et al.	78	Dasatinib	23%	10%	9%	19%	41%
Wang et al.	25	Imatinib: 24 patients Nilotinib: 1 patient	44%	175	0%	39%	0%
Hall et al.	6	1st trimester TKI use: 4 patients 1st-3rd trimester TKI use: 1 patient	17%	17%	33%	33%	0%

n: pregnancies; TKI: tyrosine kinase inhibitor.

**Table 2:** CML management during pregnancy after TKI Interruption

Study	n	Pre-Pregnancy Treatment	Therapy during Pregnancy	Outcomes
Balsat et al.	12	7 Imatinib, 3 Nilotinib, 2 None	9 PEG-IFN, 2 IFN	12 live healthy births
Law et al..	6	6 Imatinib	5 IFN, 1 Observation	1 elective abortion 5 full term deliveries
Chelyshava et al.	48	Unclear	14 Observation, 5 IFN, 1HU	14 elective abortions 1 spontaneous abortion 7 live healthy births
Alizadeh et al.	28 (16 patients)	13 Imatinib: 2 Dasatinib 1 Nilotinib	8 Observation, 8 IFN	1 spontaneous abortion 2 congenital defects 23 lives healthy births

n: pregnancies; IFN: Interferon-α; Pegylated Interferon; HU: Hydroxyurea.

## Data Availability

Not applicable.

## Supplementary Materials

The following supporting information can be downloaded at: https://www.mdpi.com/article/10.3390/hematolrep14010008/s1, Figure S1: Weeks of Pregnancy.
