# Supplementary material for: Outcomes of the Pregnancies with Chronic Myeloid Leukemia in the Tyrosine Kinase Inhibitor Era and Literature Review
Source: Hematol Rep. 2022 Mar 20;14(1):45–53. doi: 10.3390/hematolrep14010008 (PMC8953861; doi:10.3390/hematolrep14010008)
Supplement: Supplementary file 1 [file hematolrep-14-00008-s001.zip › hematolrep-1596775-supplementary.pdf]

hematology reports

Supplementary Materials: Outcomes of the Pregnancies with Chronic Myeloid Leukemia in the Tyrosine Kinase Inhibitor Era and Literature Review

Dan Ran Castillo, Daniel Park, Akhil Mehta, Simmer Kaur, Anthony Nguyen and Mojtaba Akhtari

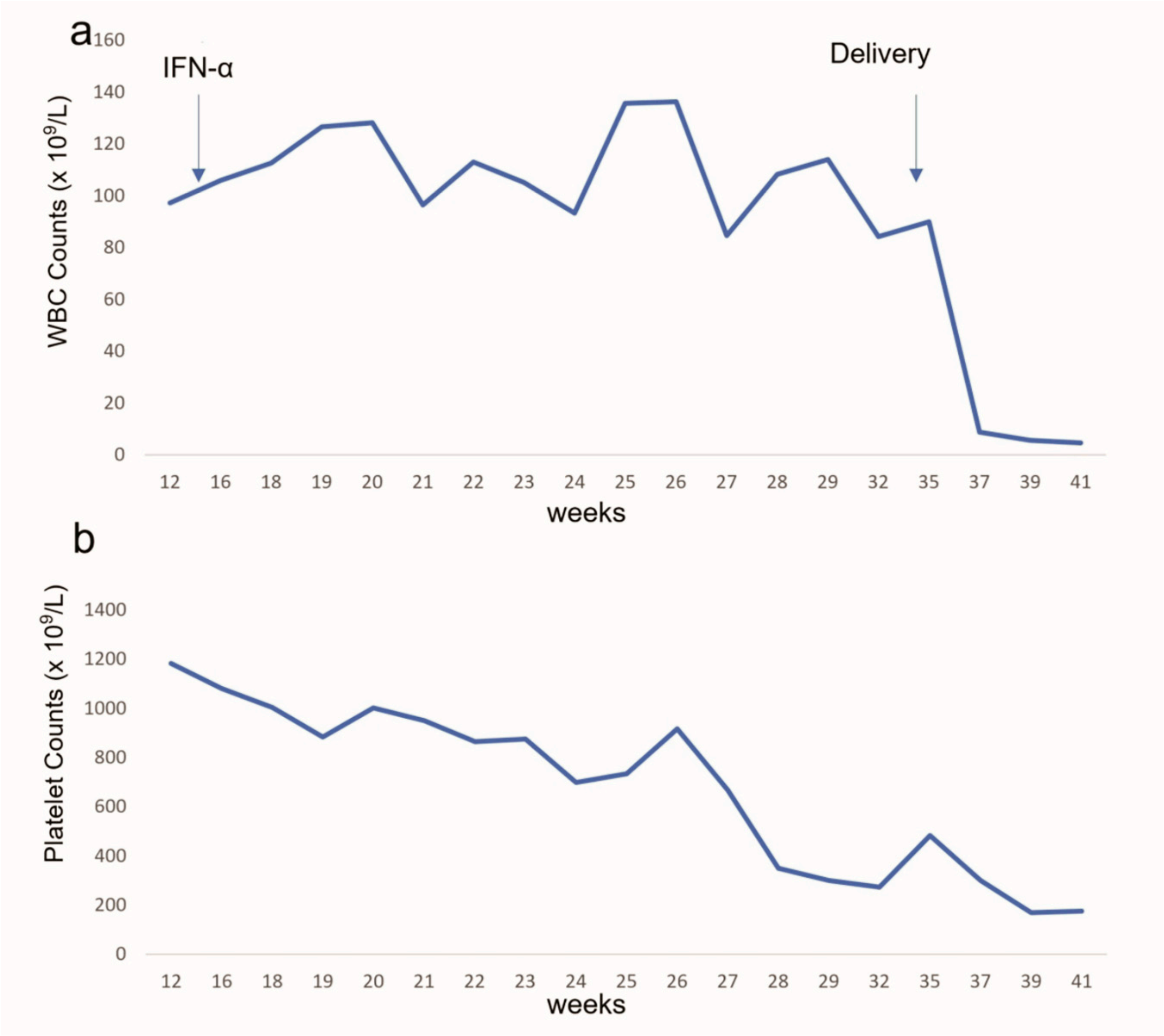

Figure S1. Weeks of Pregnancy: CBC trends while on IFN-α. a. Leukocytosis improved along with the time b. Thrombocytopenia remarkably decreased after IFN-α injection.
